# Metabolic Profiling of Breast Cancer Cell Lines: Unique and Shared Metabolites

**DOI:** 10.3390/ijms26030969

**Published:** 2025-01-24

**Authors:** Mariana Gallo, Elena Ferrari, Federica Brugnoli, Anna Terrazzan, Pietro Ancona, Stefano Volinia, Valeria Bertagnolo, Carlo M. Bergamini, Alberto Spisni, Thelma A. Pertinhez, Nicoletta Bianchi

**Affiliations:** 1 Department of Medicine and Surgery, University of Parma, 43125 Parma, Italy; mariana.gallo@unipr.it (M.G.); elena.ferrari@unipr.it (E.F.); 2 Department of Translational Medicine, University of Ferrara, 44121 Ferrara, Italy; federica.brugnoli@unife.it (F.B.); anna.terrazzan@unife.it (A.T.); pietro.ancona@unife.it (P.A.); valeria.bertagnolo@unife.it (V.B.); nicoletta.bianchi@unife.it (N.B.); 3 Department of Neuroscience and Rehabilitation, University of Ferrara, 44121 Ferrara, Italy; bgc@unife.it

**Keywords:** breast cancer, metabolite profiling, metabolome, metabolic reprogramming, NMR

## Abstract

Breast Cancer (BrCa) exhibits a high phenotypic heterogeneity, leading to the emergence of aggressive clones and the development of drug resistance. Considering the BrCa heterogeneity and that metabolic reprogramming is a cancer hallmark, we selected seven BrCa cell lines with diverse subtypes to provide their comprehensive metabolome characterization: five lines commonly used (SK-Br-3, T-47D, MCF-7, MDA-MB-436, and MDA-MB-231), and two patient-derived xenografts (Hbcx39 and Hbcx9). We characterized their endometabolomes using ^1^H-NMR spectroscopy. We found distinct metabolite profiles, with certain metabolites being common but differentially accumulated across the selected BrCa cell lines. High levels of glycine, lactate, glutamate, and formate, metabolites known to promote invasion and metastasis, were detected in all BrCa cells. In our experiment setting were identified unique metabolites to specific cell lines: xanthine and 2-oxoglutarate in SK-Br-3, 2-oxobutyrate in T-47D, cystathionine and glucose-1-phosphate in MCF-7, NAD^+^ in MDA-MB-436, isocitrate in MDA-MB-231, and NADP^+^ in Hbcx9. The unique and enriched metabolites enabled us to identify the metabolic pathways modulated in a cell-line-specific manner, which may represent potential candidate targets for therapeutic intervention. We believe this study may contribute to the functional characterization of BrCa cells and assist in selecting appropriate cell lines for drug-response studies.

## 1. Introduction

Breast cancer (BrCa) mainly affects women, with an estimated incidence of over 3 million in 2040 [1]. The treatment involves surgery, radiation, hormone modulators, chemotherapy, and immunotherapy [2]. However, depending on tumor subtype, time to diagnosis, and the presence of metastases, survival rates are still often poor. A crucial factor contributing to the poor prognosis is the intrinsic heterogeneity of BrCa, including the significant intra-phenotypic genetic variability that underlines the selection of highly aggressive clones and the development of drug resistance. BrCa classification is based on histological and molecular features that distinguish luminal A, luminal B, HER2/neu, and basal-like subtypes, which are generally paired with the expression status of the receptors for estrogen (ER), progesterone (PR), and human epidermal growth factor 2 (HER2) [3]. The total absence of those receptors defines triple-negative breast cancer (TNBC), which exhibits the greatest degree of heterogeneity and the worst clinical pattern [4,5]. In recent years, strategies to improve breast tumor stratification, particularly for TNBC, have benefited from the overlap of the immunohistochemical and molecular methods that address the expression of both polyadenylated transcripts and non-coding RNAs, single-nucleotide variants, somatic mutations, copy number variations, DNA methylation, and other epigenetic modifications [5]. In this landscape, OMICS strategies such as proteomics [6] and metabolomics [7] provide relevant data on metabolic rearrangements in BrCa, which may be useful for developing personalized treatments. It has been suggested that the serum metabolome profile could classify and predict the risk of BrCa [8]. Reprogramming of cell metabolism is becoming relevant in the search for potential inhibitors of key events in tumors, including epithelial-to-mesenchymal transition (EMT) [9], which is responsible for ineffective immune checkpoint inhibition [10] and drug resistance [11].

A comprehensive study of genetic alterations combined with metabolomic analysis has identified a panel of metabolites deregulated in ER^−^ tumors [12]. These metabolites are mainly of glycolytic and glycogenolytic origin or belong to the glutathione (GSH) pathway. In addition, specific mutations in TP53 are associated with low levels of several lipid-glycerophosphocholines [12]. As mutations and genetic aberrations induce biochemical modifications that impact pharmacologic response, cell lines showing specific genomic alterations represent valuable models for investigating the metabolic alterations induced by potential anticancer molecules. The SK-Br-3, T-47D, and MCF-7 cell lines, exhibiting distinctive cytogenetic and heterogeneous behaviors, are widely utilized as models for investigating the HER2-enriched and luminal A breast tumors [13]. It was reported that specific metabolites identified in SK-Br-3 and MCF-7 cells treated with tamoxifen/trastuzumab may be potential biomarkers to monitor the cell response to these drugs [14].

The metabolic characterization of MDA-MB-436 and MDA-MB-231 TNBC cell lines with high metastatic potential demonstrated that the glycolytic pathway and branched-chain amino acids catabolism influence cell metastatic behaviors [15]. Metabolomic analysis can be employed to evaluate the molecules that contribute to the development of drug resistance, as reported by Rushing and co-workers in their investigation on MDA-MB-231 cells exposed to doxorubicin, revealing alterations in the metabolism of arginine, proline, GSH, and β-alanine [16]. Further resources to control cancer and metastasis progression or drug resistance could be represented by the novel inhibitors of type 2 transglutaminase, as reported by Gallo et al. [17]. In this study, metabolomics has been employed to investigate the biochemical effects of these new molecules.

Since the alteration of metabolism is a hallmark of cancer cells [18,19], a better knowledge of their metabolic pathways is crucial in breast tumor research.

In this study, we conducted a metabolomic analysis on a selection of BrCa cell lines representative of the most frequent tumor phenotypes and commonly used in in vitro studies. It is estimated that approximately 60% of BrCa are ER^+^, while the remaining cases are HER2^+^ or TNBC, which are challenging to treat and investigate worldwide. Although these BrCa cell lines have been mainly characterized from a genetic and transcriptomic point of view [20], less has been done to characterize their metabolic profile. As it is recognized that BrCa cell lines still represent useful study models, their ever-improving classification by metabolomics may be relevant for further investigations. In addition, knowing that despite the considerable heterogeneity of TNBCs, in clinical practice, patients with that type of cancer typically receive the same treatment, we believe that a more precise characterization of their metabolites’ signature will facilitate the development of precision/tailored treatments. Focusing on TNBC, we included two cell lines obtained from patient-derived xenografts (PDXs) in this study. These models were selected for their enhanced capacity to mirror the intrinsic characteristics of the primary tumors [21].

We used ^1^H-NMR spectroscopy to obtain the intracellular polar metabolites profiles of SK-Br-3, T-47D, MCF-7, MDA-MB-436, MDA-MB-231, Hbcx39, and Hbcx9 BrCa cells cultured in their optimal growth conditions. This approach enabled the identification of both common and unique metabolites in cell lines exhibiting distinct proliferative and invasive properties. A comprehensive and comparative characterization of the metabolome in selected BrCa cell lines may enhance their functional analysis and contribute to a better understanding of the metabolic adaptations associated with BrCa.

## 2. Results

For this study, we selected a panel of BrCa cell lines commonly adopted in research laboratories and representative of the heterogeneity of BrCa tumors, Table 1. Focus was placed on cells with a triple-negative phenotype. We obtained the endometabolome profiles of the selected cell lines to gain insight into the metabolic characteristics associated with their diverse malignant potentials.

### 2.1. Characterization of the Main Malignant Features of BrCa-Derived Cells

**Figure 1:**
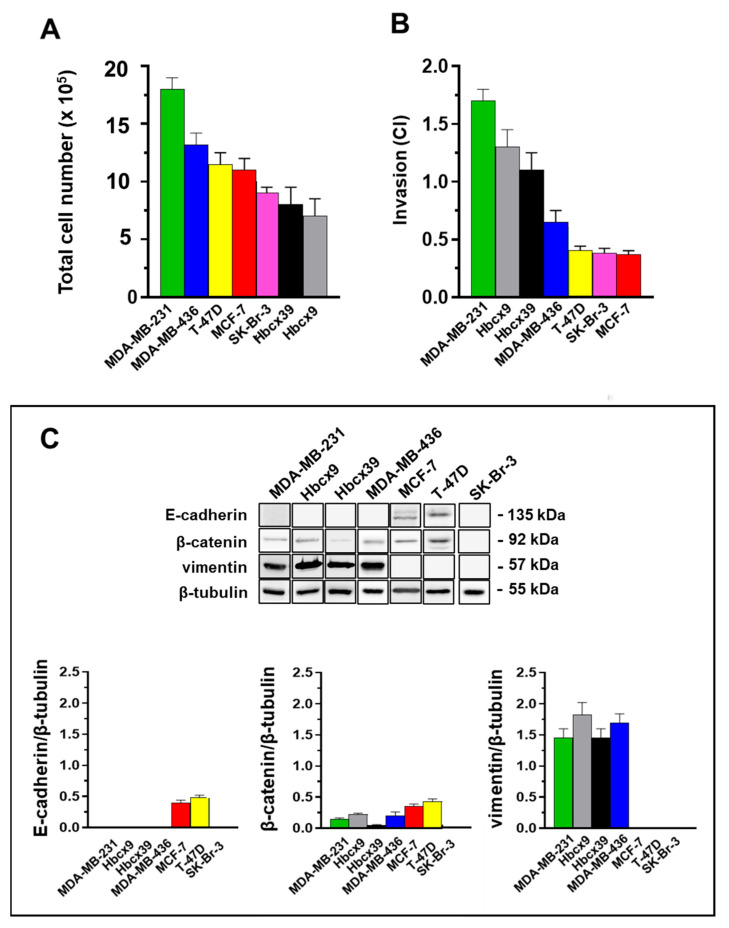
Characterization of the selected cell lines: (**A**) Proliferation assay (5 × 10^5^ cells were seeded in a sixwell plate and counted after 72 h). (**B**) Cell invasion assay. Histograms report cell index (CI) values after 24 h of impedance detection. (**C**) Western blot analysis of cell lysates. The E-cadherin, β-catenin, and vimentin levels were quantified by densitometry and normalized to β-tubulin, used as a loading control. Data are presented as mean ± standard deviation (SD) of three independent tests.

We compared the selected BrCa cell lines in terms of proliferation rate, expression of EMT markers, and invasiveness (Figure 1). Among the TNBC cells, we demonstrated that MDA-MB-231 cells display the highest proliferative and invasive potential (*p* < 0.01 for MDA-MB-231 vs. all other TNBC cell lines). In contrast, MDA-MB-436 cells show a relatively high proliferation rate but are less invasive (Figure 1A,B). The PDX-derived Hbcx9 and Hbcx39 cell lines exhibit a reduced growth rate compared to MDA-MB-436 cells (*p* = 0.007 and *p* = 0.02, respectively) (Figure 1A). However, they display enhanced invasive properties (*p* = 0.005 and *p* = 0.02, respectively) (Figure 1B).

The four selected TNBC cell lines express similar levels of the mesenchymal marker vimentin, varying amounts of β-catenin, and lack E-cadherin expression (Figure 1C). Consistent with their luminal A phenotype [22], the T-47D and MCF-7 cells express E-cadherin and β-catenin, but not vimentin (Figure 1C). They reveal similar and intermediate growth rates (Figure 1A) and the lowest invasive potential (Figure 1B). Concerning the HER2-enriched SK-Br-3 cells, they show growth and invasive potential similar to luminal A cells (*p* > 0.05), without detectable levels of EMT markers in our experimental conditions (Figure 1A–C).

### 2.2. BrCa Cell Lines Metabolite Profiling

We set up three independent replicates of each cell line to define their metabolic profiles under favorable growth conditions. The ^1^H-NMR analysis aimed to quantify their major metabolites and to identify the active metabolic pathways.

**Figure 2:**
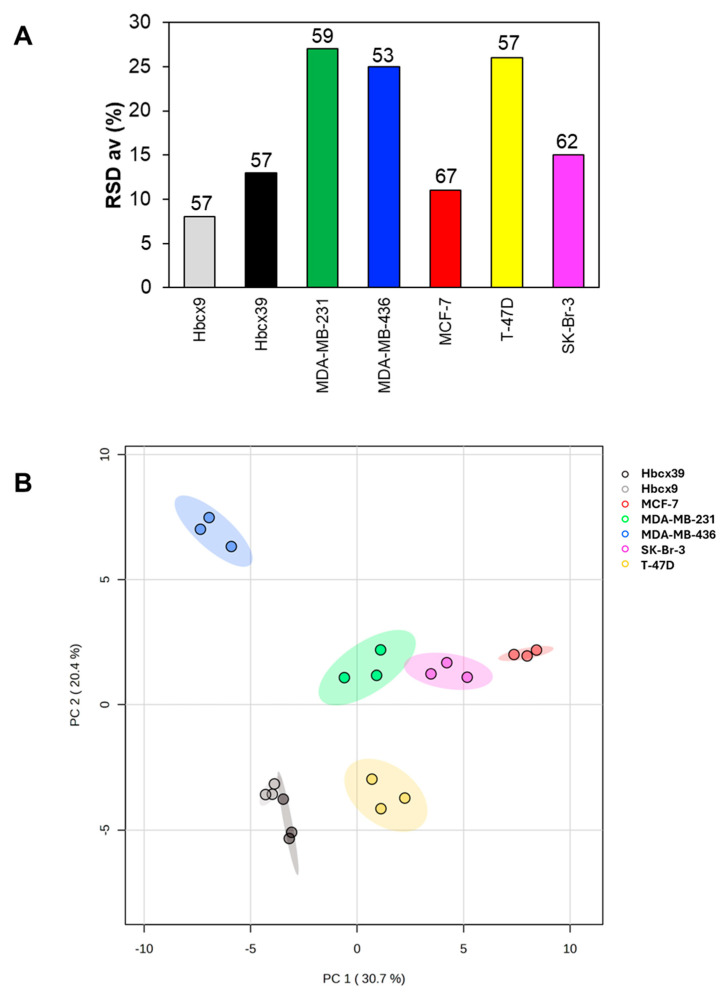
(**A**) Analysis of metabolite concentration datasets. The average relative standard deviation (RSD_av_) for all the measured metabolite concentrations indicates the reproducibility among replicates (n = 3). The numerical value associated with each bar represents the number of identified metabolites. (**B**) Principal component analysis (PCA) scores plot for the metabolite datasets. PC, principal component. For each metabolome cluster, the ellipse represents a 95% confidence interval.

High-quality ^1^H-NMR spectra of the BrCa intracellular extracts (Figure S1) allowed the identification and quantification of 62 metabolites in SK-Br-3, 57 in T-47D, 67 in MCF-7, 53 in MDA-MB-436, 59 in MDA-MB-231, and 57 in both Hbcx39 and Hbcx9 cells. The quality of the datasets is reflected by the average relative standard deviation (RSD_av_) of the triplicates (Figure 2A), ranging from 8 to 27%. The MDA-MB-436, MDA-MB-231, and T-47D cells exhibited more variable metabolite concentrations among the triplicates, confirming the heterogeneity reported in the literature [20,23,24].

Principal component analysis (PCA), an unsupervised method, was applied to the seven datasets obtained, providing well-separated clusters representing the cell types and reflecting their distinct metabolome compositions (Figure 2B). Interestingly, the samples from the PDX-derived cells (Hbcx39 and Hbcx9) produced compact and separate clusters confirming, at the metabolite level, the high stability of this experimental model [25].

**Figure 3:**
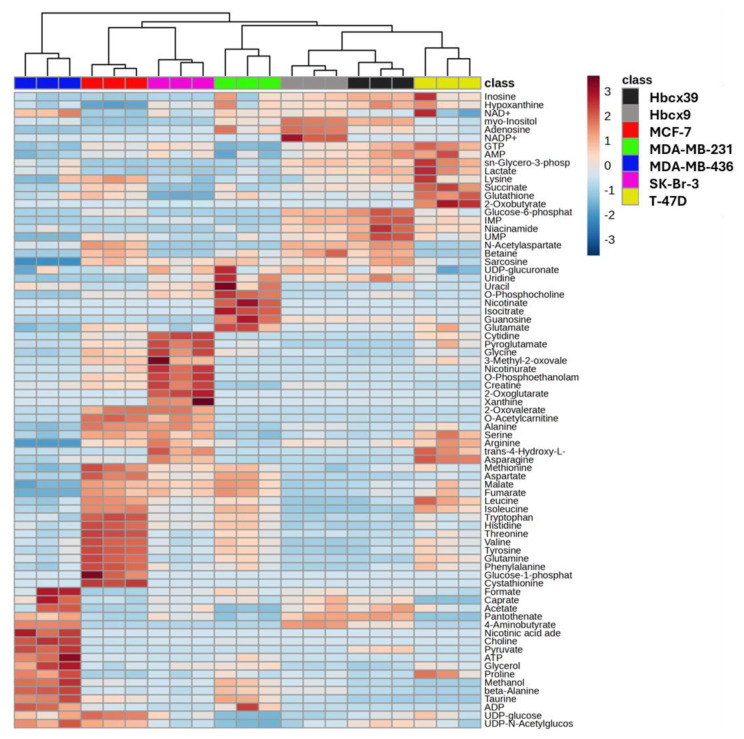
Heatmap and dendrogram of metabolite concentration datasets. Sample triplicates are presented in columns, with the metabolites displayed in rows. The relative abundance is represented by color code (blue, lower abundance; red, higher abundance). Samples are separated using hierarchical clustering (Complete’s algorithm), with the resulting dendrogram scaled to represent the distance between each branch (Euclidean distance measure).

The heatmap (Figure 3) displays the relative expression level of the metabolites in all cell replicates, demonstrating that each BrCa cell line exhibits a distinct metabolite pattern, reflecting differences in metabolites’ composition and concentration. In the hierarchical clustering dendrogram, Euclidean distance metrics provide evidence of the close relationship between Hbcx39 and Hbcx9. Moreover, the T-47D metabolome composition is clustered with the triple-negative MDA-MB-231 and PDX-derived cells, while SK-Br-3 is grouped with MCF-7 cells. Interestingly, the MDA-MB-436 cell line exhibits the greatest divergence from the other TNBC cells (Figure 3).

### 2.3. Metabolites Common to the Panel of BrCa Cell Lines

**Figure 4:**
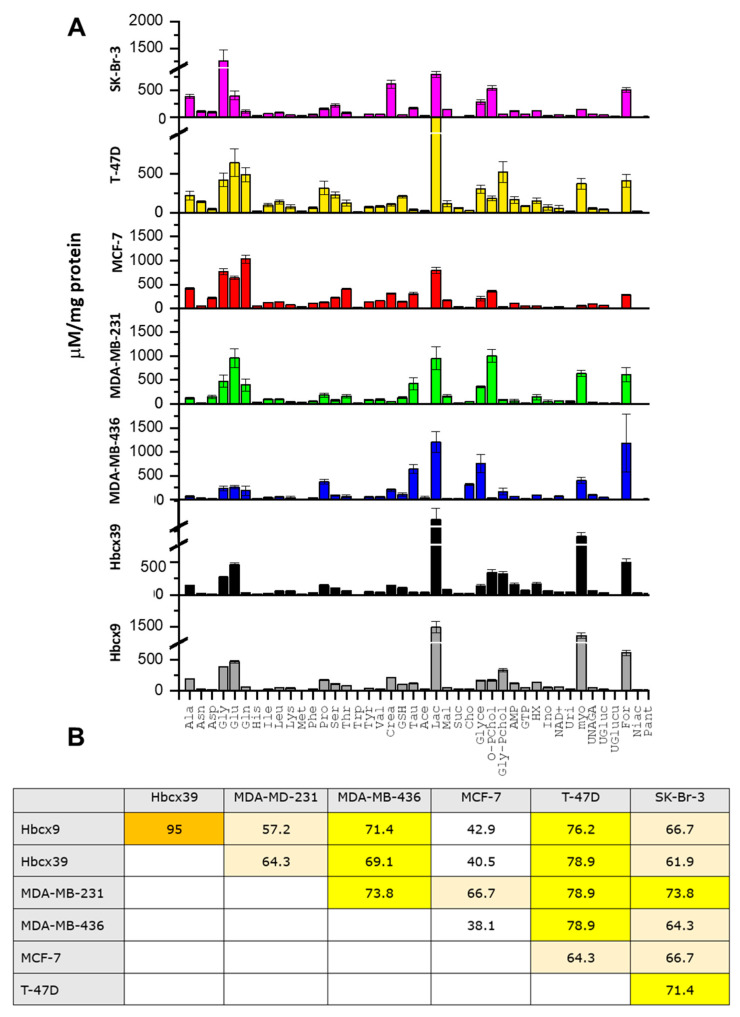
Concentration analysis of the common metabolites: (**A**) Profiles of normalized (by protein content) and auto-scaled concentrations (μM/mg protein) of the 42 common metabolites. (**B**) Pairwise comparison matrix displaying the similarity percentages. These percentages were calculated considering the number of metabolites with similar concentrations (fold changes < 2 and *p*-values > 0.01) as determined by volcano plot analyses of the 42 common metabolites (see M&M). The yellow shades of the boxes indicate the degree of pairwise similarity: the darker, the greater the similarity. Ace, acetate; Cho, choline; Crea, creatine; For, formate; Glyce, glycerol; Gly-PChol, glycerophosphocholine; GSH, reduced glutathione; HX, hypoxanthine; Ino, inosine; Lac, lactate; Mal, malate; myo, myoinositol; Niac, niacinamide; O-PChol, O-phosphocholine; Pant, pantothenate; Suc, succinate; Tau, taurine; UGluc, UDP-glucose; UGlucu, UDP-glucuronate; UNAGA, UDP-N-acetylglucosamine; Uri, uridine.

A comparative analysis of the metabolites detected in all the selected cell lines revealed 42 common metabolites, differently accumulated in the seven BrCa cells (Figure 4A). The number of metabolites exhibiting comparable concentrations in paired cell line profiles was used to calculate their “similarity percentage” (see the M&M section), as illustrated in the pairwise comparison matrix in Figure 4B. The two PDX-derived cell lines exhibit the highest similarity percentage (95%). The PDX-derived cells reveal a higher degree of similarity with the MDA-MB-436 cells (69.1% and 71.4% and with Hbcx39 and Hbcx9, respectively) than the MDA-MB-231 cells (64.3%and 57.2%), showing a divergent character of the latter compared to the other TNBC cells. Surprisingly, even if T-47D and MCF-7 present the same phenotype (ER^+^/PR^+^/HER2^−^), T-47D cells share more metabolites with TNBCs (76.2–78.9%) than with MCF-7 cells (64.3%). SK-Br-3 cells (ER^−^/PR^−^/HER2^+^) share more metabolites with T-47D cells (71.4%) and MDA-MB-231 (73.8%) compared to the other cells. The lowest similarity percentages are observed for the MCF-7 cells. They exhibit a limited degree of similarity to the SK-Br-3, T-47D, and MDA-MB-231 cells (66.7%, 64.3%, and 66.7%, respectively), and lower values, below 43%, are found when compared to the other TNBCs.

Figure 4A illustrates the concentrations of the 42 common metabolites intermediates of shared metabolic pathways. All cell lines showed high levels of glycine, glutamate, lactate, and formate (>250 μM/mg protein). Other metabolites demonstrated differential enrichment at lower concentrations.

The higher levels of glutamine, an amino acid added at the same concentration in the culture medium of all cells, were observed in SK-Br-3, T-47D, MCF-7, and MDA-MB-231; the lower ones were measured in Hbcx cells. Other amino acids were found to be accumulated in non-TNBC phenotypes. The higher concentrations of serine were found in SK-Br-3, T-47D, and MCF-7, cells; glycine and alanine were preferentially concentrated in SK-Br-3 and MCF-7 cells. MCF-7 also exhibits high levels of creatine, a metabolite derived from glycine involved in energy transfer. Higher levels of threonine were observed in MCF-7; proline was detected at a high concentration in T-47D and MDA-MB-436 cells, characterized by a consistent secretion of extracellular matrix components [26].

All TNBC cell lines exhibited high levels of myo-inositol. High levels of both myo-inositol and taurine distinguished MDA-MB-436 and MDA-MB-231 cell lines from PDX-derived cells. A relatively high concentration of myo-inositol was also observed in T-47D cells.

BrCa cells are characterized by high expression of glucose transporters and enzymes involved in glucose metabolism, a fact that affects the prognosis and evolution of cancer [27]. In our culture conditions, glucose is a non-limiting component of the media, with concentrations of 3 g/L for SK-Br-3, 2 g/L for T-47D, and 4.5 g/L for all other cell lines. Nevertheless, we observed that the intracellular concentration of lactate, the major product of energetic metabolism, is higher in the T-47D and TNBCs cells than in the other lines.

Choline metabolism deserves special mention because it is highly dysregulated in multiple cancers, including BrCas [28]. The levels of choline compounds in the selected cell lines are illustrated in Figure S2. The total choline content of MDA-MB-231 cells is markedly elevated compared to the other cells that exhibit comparable levels. The highest concentration of choline was found in MDA-MB-436 cells, while the higher levels of O-phosphocholine were observed in SK-Br-3, MCF-7, and MDA-MB-231 cells, despite their lower choline and glycerophosphocholine content (Figure 4A and Figure S2). In contrast, the T-47D and PDX-derived cells showed the highest levels of glycerophosphocholine (Figure 4A and Figure S2).

### 2.4. Exclusive and Enriched Metabolites in BrCa Cells

The sensitivity of our method [29] and the culture conditions adopted allowed the identification of exclusive metabolites for each cell line profile, except for Hbcx39, which exhibited a metabolome composition practically identical to that of Hbcx9, except for NADP^+^. The unique metabolites are listed in Table 2: xanthine and 2-oxoglutarate are present exclusively in the SK-Br-3, 2-oxobutyrate in the T-47D; cystathionine and glucose-1-phosphate in the MCF-7; NAD^+^ in the MDA-MB-436; isocitrate in the MDA-MB-231; and NADP^+^ in the Hbcx9 profiles.

The metabolic pathway analysis, performed considering the exclusive (Table 2) and the enriched metabolites (heatmap scores >1 in Figure 3 and listed in Table S1), indicates the pathways preferentially modulated in a cell-line-specific manner. Diverse pathways are highlighted in each of the four TNBC cell lines: nicotine and niacinamide in MDA-MB-436, the Krebs cycle and the metabolism of pyrimidine in MDA-MB-231, purine in Hbcx39, and inositol phosphate in Hbcx9. Note that the enriched metabolites AMP, hypoxanthine, IMP, and GTP, which are involved in purine metabolism, are shared by T-47D and PDX-derived cell lines. The pathway analysis of the SK-Br-3 and MCF-7 cell lines reports alterations in amino acid metabolisms. As for SK-Br-3 cells, the metabolism of glycine, serine, threonine and arginine biosynthesis are altered. Finally, the MCF-7 cell line exhibits modification of the phenylalanine, tyrosine, and tryptophan biosynthesis pathway and sulfur-containing amino acid metabolism.

## 3. Discussion

The current classification of BrCas does not adequately account for their extensive phenotypic heterogeneity, thus hampering a correct diagnosis [3,23,24]. To ameliorate their characterization, we have conducted a comparative metabolomic study on a panel of cell lines representative of the most frequent tumor subtypes, identified based on the current histopathological and molecular classifications: namely, the SK-Br-3 cell line, showing HER2-enriched phenotype (15–20% of tumors), and the T-47D and MCF-7 cell lines, belonging to the luminal A phenotype (which account for 50–60% of breast tumors) [1]. These cell lines do not reflect substantial differences in terms of invasion, even though their phenotypes are characterized by different EMT marker expressions (Figure 1). The selection also includes four cell lines representative of the most aggressive TNBC tumors (15–20% of BrCas), lacking hormone receptors and targeted therapies, and frequently associated with TP53 and/or BRCA alterations [5,30,31,32]. We investigated the well-known MDA-MB-436 and MDA-MB-231 cell lines [33] and two PDX-derived cell lines, Hbcx39 and Hbcx9 [34,35]. These last two cell lines belong to a different molecular subtype [36,37] and exhibit a distinct panel of genomic alterations (Table 1).

Although all the selected TNBC cell lines were E-cadherin-negative and vimentin-positive, they showed significantly different proliferation rates (with MDA-MB-231 > MDA-MB-436 > PDX-derived cell lines) and invasive potential (with MDA-MB-231 > PDX-derived cell lines > MDA-MB-436). As expected, TNBC cell lines revealed an invasive potential higher than the other phenotypes. In contrast, the proliferation rate of PDX-derived cell lines was lower than those of the selected luminal A and HER2-enriched cells (Figure 1). Indeed, our analysis confirms that the histopathological/molecular classification alone is not entirely predictive of the malignant potential of breast tumor cells, which is expressed in terms of aggressive and metastatic behavior.

It has been proposed that knowledge of the metabolic profiles of TNBCs may help to identify additional markers [38], which could potentially represent novel therapeutic targets for BrCa [39].

Our analyses demonstrate that the selected cell lines show well-defined metabolite patterns and agree with the data reported by Dubuis and collaborators [40]. As expected, PCA distinguishes the cell lines according to their metabolomes (Figure 2B). The heatmap analysis displays a distinct pattern of metabolite levels for each cell line (Figure 3). Interestingly, despite their genetic differences, PDX-derived cell lines revealed similar metabolite composition and low variability in metabolite concentrations. This finding supports the hypothesis that PDX-derived cells are a more suitable model for testing potential anticancer agents [21].

Despite their different metabolic profiles, the accumulation of glycine, glutamate, lactate, and formate is evident across all metabolite profiles (Figure 4B). The high lactate levels are indicative of the use of glycolysis and the Warburg effect, with the highest levels observed in TNBC and T-47D cell lines. The role of formate in the transition between normal and pathological conditions is a major topic in tumor investigations [41]. Furthermore, formate has been associated with invasiveness enhancement and the spread of metastasis linked to lipid metabolism [42]. In several BrCa cell lines, reduction in the expression of the enzyme that produces NADPH by catabolizing 10-formyltetrahydrofolate into CO_2_ and tetrahydrofolate, the aldehyde dehydrogenase 1 family member L2, increases ROS levels and the production of formate and formyl-methionine levels, which correlated with enhanced migration capacity [43]. In our experiments, consistently, we observe a higher accumulation of formate, especially in cells with a greater propensity to invasion and expressing vimentin (TNBCs) (Figure 1 and Figure 4B). In a BrCa mouse model, formate was associated with increased serine catabolism and mitochondrial oxidative metabolism [44]. Thus, formate represents an interesting metabolite for its involvement in folate and 1C metabolism, on which to focus studies in BrCa models.

Our results show a ratio [glutamate]/[glutamine] ranging from 1.4 to 11.7 in the TNBC cell lines, which is higher than the ratio observed in T-47D (1.3) and MCF-7 (0.6) cells. These data support an enrichment in glutamate associated with the ER-status and negatively correlated with the peroxisome proliferator-activated receptor pathway, patient survival, and sensitivity to glutaminase inhibitors [45].

Choline metabolism is a hallmark of cancer [46] and could be employed for diagnosis [47,48]. Its metabolism controls the composition of lipids in the plasma membrane, the structural integrity of which influences communication between cells and trans-membrane signaling [49]. Choline is linked to betaine and methionine–homocysteine cycle by methylation processes and trans-sulfuration pathway with GSH production [50]. MDA-MB-231, the most proliferative and invasive cell line (Figure 1), exhibited the highest total choline level, mainly due to its high O-phosphocholine content. O-phosphocholine was also the most concentrated choline-derived metabolite in SK-Br-3 and MCF-7 cell lines. In BrCa, a high concentration of O-phosphocholine, depending on its increased transport and choline kinase activity, represents a relevant marker [51]. On the other hand, high levels of glycerophosphocholine, which characterize both PDX-derived and T-47D cells, correlate to basal-like tumors in xenografts [52].

We identified exclusive metabolites for each cell line in our experimental setting, potentially representing pharmacological targets. Indeed, in the most aggressive MDA-MB-231 cells, we found a high concentration of isocitrate, an intermediate of the Krebs cycle. The knockdown of the isoform 1 of isocitrate dehydrogenases (IDH1), an enzyme that oxidatively decarboxylates isocitrate to α-ketoglutarate, could accelerate the migration and invasion ability of these cells through the increase in EMT regulators, such as Snail and Slug, affecting mitogen-activated protein kinase–hypoxia-inducible factor-1 alpha/nuclear factor kappa B signaling [53]. Liu and colleagues also reported that a low expression level of IDH1 significantly correlated to advanced-stage lymph node metastasis and poor disease survival. Furthermore, a multivariate regression analysis revealed that low IDH1 and high Snail expression may constitute a potential prognosis biomarker in patients with BrCa [53].

On the other hand, Li and colleagues [54] focused on wild-type IDH2 as a therapeutic target in TNBC. They examined the effect of wild-type IDH2 inhibition and observed an increase in α-ketoglutarate level, suggesting a suppression of the Krebs cycle. The accumulation of this intermediate led to a reduction in glycolysis and ATP depletion, consequently inhibiting cell proliferation and EMT and correlating with the small size of the tumors in MDA-MB-231 xenografts.

We also found the pyrimidine pathway to be enriched in MDA-MB-231 cells (Table 2), suggesting a key role in these cells. In line with this, Liao and coworkers [55] described the effects of pyrimidine biosynthesis inhibitor treatment (brequinar) in combination with a glutaminolysis inhibitor (CB-839), targeting these pathways in multiple TNBC models: TNBC cell lines and PDX, in in vitro and in vivo experiments. In vivo, they found a therapeutic efficacy: decreased tumor size and improved survival.

NAD^+^ is particularly elevated in MDA-MB-436 cells. Its concentration may be determined by the increased expression of NAD^+^ biosynthetic enzymes, impacting the tumor microenvironment [56]. Since NAD^+^ biosynthesis is mainly sustained by nicotinamide phosphoribosyl transferase, NAD^+^ depletion induced by inhibitors of this enzyme can be an effective strategy in cancer therapies [57]. Differently, the Hbcx9 cells, which have been reported to be more responsive than Hbcx39 to cisplatin [34], exhibited an elevated concentration of NADP^+^, a pivotal coenzyme in lipid biosynthesis and regulation of ROS levels. De novo synthesis of NADP^+^ is catalyzed by NAD^+^ kinase (NADK) and upregulated in the BrCa tumors with more metastatic potential [58]. NADK inhibitors could represent clinically relevant drugs in cancer therapy, such as the lead compound thionicotinamide, an NADK inhibitor prodrug already assayed on human B-cell lymphoma and colon cancer xenograft mice [59]. All other metabolites are common to PDX-derived cells, contributing to a similarity of 95% (Figure 4B).

Metabolic pathways analysis based on the enriched and exclusive metabolites of Hbcx39 and Hbcx9 cells identified the inositol phosphate and purine metabolisms, respectively. Concerning phosphate metabolism, inositol 1, 4, 5 trisphosphate receptor type 2 and type 3 increased in tumors compared to adjacent normal tissues in patients affected by BrCa [60].

The relevance of purine metabolism also emerged in a multi-omics study [38] focused on TNBC phenotype, analyzing samples from patients, xenograft models, and cell lines, including MDA-MB-346 and MDA-MB-231. Based on metabolomics data, the authors characterized three different metabolic-pathway-based subtypes (MPSs) of TNBCs: MPS1, MPS2, and MPS3. The MPS2 resulted in the more aggressive and invasive, showing high levels of carbohydrates and purine and pyrimidine metabolism, a feature displayed by our cell lines. The MPS2 subtype correlated with a lower relapse-survival time and carried *TP53* mutations. Cells with MPS2 profile responded to therapy with lactate dehydrogenase inhibitors. In this regard, our TNBC and PDX-derived cells could represent good candidates for further in vitro drug investigations.

In T-47D cells, we detected 2-oxobutyrate, a product of the catabolism of sulfur-containing amino acids by the methionine gamma-lyase, which has already been suggested as a potential therapeutic target in several cancers [61]. In MCF-7 cells, we observed the accumulation of glucose-1-phosphate and cystathionine. The former has regulatory functions associated with EMT [62], and the latter is an oncometabolite in BrCa [63]. In SK-Br-3 cells, with ER^−^ and HER2^+^ phenotypes, we detected elevated concentrations of 2-oxoglutarate and xanthine, which emerge as potential metabolic markers. Xanthine, metabolized by xanthine oxidoreductase, is fundamental for the physiological cell differentiation of mammary gland epithelium, and its loss has been associated with a short time of disease-free life in patients affected by breast tumors [64,65]. Furthermore, a metabolomic analysis performed on tamoxifen-resistant MCF-7 revealed a significant increase in the levels of xanthine and a down-regulation of xanthine dehydrogenase and glutathione synthase gene expression compared to tamoxifen-sensitive cells. This metabolic change correlates with a poor outcome for BrCa patients, suggesting a possible role of these molecules in the development of resistance [64]. It must be emphasized that 2-oxoglutarate regulates the activity of its dependent dioxygenases, which are responsible for epigenetic modifications [66]. Indeed, a 2-oxoglutarate-dependent oxygenase acting as a proline hydroxylase was positively associated with proliferation and poor survival in TNBC cells [67].

In conclusion, our investigations demonstrate that each cell line, despite its histopathological/molecular phenotype, exhibits a distinctive metabolic profile. The identification of differentially accumulated metabolites provides a rationale to pin down metabolic pathways suitable as targets for the design of new drugs. This hypothesis is of particular significance in the context of highly heterogeneous BrCas.

## 4. Materials and Methods

### 4.1. Cell Lines

We performed cultures of MCF-7 (RRID: CVCL_0031), T-47D (CVCL_0553), SK-Br-3 (CVCL_0033), MDA-MB-231 (RRID: CVCL_0062), and MDA-MB-436 (CVCL_0623) BrCa cell lines, purchased from American Type Culture Collection (Manassas, VA, USA). The Hbcx9 and Hbcx39 cell lines, established from PDXs of TNBC, were provided by Xentech (Evry, France). After thawing, the cells were grown and amplified for no more than twelve passages. Mycoplasma assay (MERCK, Darmstadt, Germany) was carried out monthly.

Table 1 describes the employed cell lines’ phenotypes and primary genetic mutations. MCF-7 and T-47D (luminal A) are ER^+^, PR^+^, and HER2^−^, with low levels of the Ki-67 proliferation marker; SK-Br-3 is HER2-enriched, with high levels of Ki-67. MDA-MB-231, MDA-MB-436, Hbcx9, and Hbcx39 show a TNBC (ER^−^/PR^−^/HER2^−^) phenotype. We verified the maintenance of genetic features quarterly by single nucleotide polymorphism analysis.

### 4.2. Cell Culture Conditions

We cultured the different cell lines using specific media supplemented with 2 mM glutamine, 50 U/mL penicillin, and 50 μg/mL streptomycin (Merck Life Science, Milan, Italy). For MCF-7, MDA-MB-436, and MDA-MB-231 cells, Dulbecco’s Modified Eagle Medium high glucose (Gibco™, Thermo Fisher Scientific, Monza, Italy) was used; SK-Br-3 was cultured in McCoy’s 5a Medium Modified (Gibco™), and T-47D cells were grown in RPMI-1640 Medium (Gibco™). All media were supplemented with 10% fetal bovine serum (FBS, Thermo Fisher Scientific, Inc.). As previously reported [68], the Hbcx9 and Hbcx39 established cell lines were cultured in Gibco™ Advanced DMEM-F12 with 8% FBS.

Aware of glucose’s crucial role in BrCa cell growth [69], we used an appropriate glucose concentration for each cell line, within the 2.0 to 4.5 g/L range.

Cells were seeded at the concentration 4 × 10^5^ cells/mL, and cultures were carried out at 37 °C in a 5% CO_2_ humidified atmosphere in T125 flasks until reaching a maximum of 70–80% confluence. All experiments were performed in triplicate.

### 4.3. Real-Time Assay of Cell Invasion

All cell lines were analyzed for their invasion capabilities by xCELLigence RTCA system (Real-Time Cell Analyzer System, Acea Biosciences Inc., San Diego, CA, USA), as previously reported [70,71]. The CIM-16 plate upper chamber was layered with diluted Matrigel (1:20 in serum-free medium, BD Biosciences, Milan, Italy), and the bottom chamber was filled with medium containing 10% FBS as a chemoattractant. About 4 × 10^5^ cells/well were applied directly on the Matrigel, and each determination was performed in triplicate, with signal detection every 15 min for 24 h. Impedance values were expressed as a dimensionless parameter (CI), and values greater than 0.1 were considered positive.

### 4.4. Immunochemical Analysis

Whole-cell lysates were separated on 7.5% polyacrylamide denaturing gels (SDS-PAGE) and blotted to nitrocellulose membranes (GE Healthcare Life Science, Little Chalfont, UK). After blocking residual reactive groups, the membranes were exposed to antibodies directed against E-cadherin, β-catenin, vimentin, and β-tubulin as previously reported [72]. All antibodies were purchased from Santa Cruz Biotechnology (Santa Cruz, CA, USA). The immunocomplexes were detected by chemiluminescence using the ECL system (PerkinElmer, Boston, MA, USA). The chemiluminescence-derived bands were acquired with the ImageQuantTM LAS 4000 biomolecular imager (GE Healthcare Life Science). The densitometric analysis was performed using ImageQuant TL software v. 7.0 (GE Healthcare Life Science).

### 4.5. Collection of Intracellular Metabolites

After detachment by incubation with 4 mL trypsin-EDTA 0.25% for 2 min at 37 °C (Thermo Fisher Scientific, Monza, Italy) and inactivation of trypsin with 10% FBS, cells were collected by centrifugation at 300× *g* for 10 min at 4 °C. Finally, cells were washed three times with 10 mL of phosphate-buffered saline pH 7.4 (PBS, Thermo Fisher Scientific). After a final centrifugation step, pellets were stored at −80 °C until use.

Intracellular metabolites were obtained as previously described [17]. Briefly, cell pellets of each cell line were thawed, suspended in 4 mL of deionized water at 4 °C, and vortexed. Cell lysis was achieved by two cycles of 30 sec sonication of the cell suspensions immersed in an ice bath. Fifty microliters of each lysate was collected for protein quantification (Bradford assay, Sigma-Aldrich, Merck KGaA, St. Louis, MI, USA). The lysates were ultra-filtered using Amicon Ultra-4 Centrifugal filters (10,000 MWCO, Merck Millipore, Milan, Italy) at 4000× *g* and 5 °C to deplete cell debris and proteins [73]. The filtered metabolite solutions were lyophilized and stored at −80 °C until NMR spectra acquisition.

### 4.6. Metabolomics Analysis

#### Sample Preparation and ^1^H-NMR Spectra Acquisition

The lyophilized metabolite samples were dissolved in 600 μL of phosphate buffer (50 mM pH 7.4), containing 2.5% D_2_O for the signal lock and 1.45 mM 3-trimethylsilyl propionic acid as chemical shift reference and quantitative internal standard. The ^1^H-NMR spectra were acquired at 25 °C in a JEOL 600 MHz ECZ600R spectrometer (JEOL Inc., Tokyo, Japan) using the first increment of NOESY presat pulse sequence, 128 scans, according to Gallo and co-workers [74]. The spectra were processed with zero-filling to 256 k data points and line broadening of 0.5 Hz and analyzed using Chenomx NMR suite 9.0 software (Chenomx Inc., Edmonton, AB, Canada).

### 4.7. Statistical Analysis

Data describing cell proliferation, invasion, and the expression of EMT markers are presented as the mean ± SD of three independent replicates for each cell line. Multiple comparisons were performed using analysis of variance (ANOVA) with Bonferroni correction to test for significant differences between the means of cell proliferation and invasion data. A two-tailed *t*-test with unequal variances was employed to analyze pairwise differences. A *p*-value < 0.05 was considered statistically significant.

Multivariate statistical analysis was performed on target metabolites using MetaboAnalyst 6.0 (https://www.metaboanalyst.ca on 11 January 2024) [75,76]. Metabolite concentrations were normalized by the protein cell content of each sample and auto-scaled (mean-centered and divided by the standard deviation of each variable) before analysis.

PCA with 95% confidence ellipses was employed as an exploratory data analysis tool. The heatmap analysis compared the metabolite profiles of BrCa cell lines using the Euclidean distance measure and the “complete clustering” mode.

The percentage of “metabolic similarity” between pairs of cell types was derived from the volcano plots comparing the concentrations of common metabolites. Volcano plots typically identify metabolites that exhibit meaningful concentration differences between two metabolite profiles. In our study, we applied volcano plots with a different approach: to identify, among the 42 common metabolites detected in all cell lines, those with similar concentrations, i.e., with a fold change < 2 and a *p*-value > 0.01. To illustrate the degree of metabolic similarity between the BrCa cell lines, we generated a pairwise comparison matrix reporting the percentage of the 42 common metabolites exhibiting comparable concentrations in cell line profiles.

## Figures and Tables

**Table 1:** Characteristics of the employed BrCa cell lines.

Cell Type	Receptor Status			Subtype	Clinical Features of Derived Tumor	Mutation/ Deletion (Census Tier 1) ^a^
	ER	PR	HER2			
SK-BR-3	−	−	+	HER2^+^	AC	*CDH1* *TP53*
T-47D	+	+	−	LA	IDC	*PIK3CA* *SPEN* *TP53* *ARID1A* *ACVR1* *AFDN* *KDM5C* *KMT2C*
MCF-7	+	+	−	LA	IDC	*ERBBA* *PIK3CA* *GATA3*
MDA-MB-436	−	−	−	TNA (Lehmann’s classification: M)	AC	*BRCA1* *RB1* *TP53*
MDA-MB-231	−	−	−	TNB (Lehmann’s classification: M)	AC	*BRAF* *CD79A* *CRTC3* *CDKN2* *KRAS* *PDGFRA* *NF2* *TP53*
Hbcx39	−	−	−	TNBC Lehmann’s classification: BL1	IDC	*TP53* *KRAS*
Hbcx9	−	−	−	TNBC Lehmann’s classification: BL1	IDC	*ATM* *CDH1* *TP53*

^a^ Tier 1 classification identifies mutations relevant to oncogenic transformation. AC, adenocarcinoma; BL1, basal-like 1; ER, estrogen receptor; HER2, human epidermal growth factor receptor 2; IDC, invasive ductal carcinoma; LA, luminal A; PR, progesterone receptor; TNA, triple-negative A; TNB: triple-negative B; TNBC, triple-negative breast cancer.

**Table 2:** Cell line-specific metabolites and pathway analysis.

Cell Line	Input ^a^	Match	*p*-Value	Exclusive Metabolites ^b^	Pathway
SK-Br-3	15	3/33 2/14	0.001 0.023	Xanthine 2-Oxoglutarate	Glycine, serine, and threonine metabolism Arginine biosynthesis
T-47D	14	3/70	0.017	2-Oxobutyrate	Purine metabolism
MCF-7	22	3/33 2/4	0.006 0.008	Cystathionine Glc-1P	Cysteine and methionine metabolism Phenylalanine, tyrosine, and tryptophan biosynthesis
MDA-MB-436	17	2/15	0.009	NAD^+^	Nicotinate and nicotinamide metabolism
MDA-MB-231	9	2/20 2/39	0.004 0.015	Isocitrate	TCA cycle Pyrimidine metabolism
Hbcx39	12	4/70	0.001	-	Purine metabolism
Hbcx9	9	2/30	0.011	NADP^+^	Inositol phosphate metabolism

^a^ The metabolite entries, listed in Table S1, were derived from the heatmap analysis (mean scores > 1). ^b^ Cell-line-specific metabolite/s.

## Data Availability

Data will be made available on request.

## Supplementary Materials

The following supporting information can be downloaded at: https://www.mdpi.com/article/10.3390/ijms26030969/s1.
