# Supplementary material for: Metabolic Profiling of Breast Cancer Cell Lines: Unique and Shared Metabolites
Source: Int J Mol Sci. 2025 Jan 24;26(3):969. doi: 10.3390/ijms26030969 (PMC11816582; doi:10.3390/ijms26030969)
Supplement: Supplementary file 1 [file ijms-26-00969-s001.zip › ijms-3401295-supplementary.docx]

**Metabolic profiling of breast cancer cell lines: unique and shared metabolites**

Mariana Gallo ^1#^, Elena Ferrari ^1#^, Federica Brugnoli ^2^, Anna Terrazzan ^2^, Pietro Ancona ^2^, Stefano Volinia ^2^, Valeria Bertagnolo ^2^, Carlo M. Bergamini ^3^, Alberto Spisni ^1*^, Thelma A. Pertinhez ^1^*, Nicoletta Bianchi ^2^

^1^ Department of Medicine and Surgery, University of Parma, Parma, Italy; MG: [mariana.gallo@unipr.it](mailto:mariana.gallo@unipr.it); EF: [elena.ferrari@unipr.it](mailto:elena.ferrari@unipr.it); AS: [alberto.spisni@unipr.it](mailto:alberto.spisni@unipr.it); TAP: [thelma.deaguiarpertinhez@unipr.it](mailto:thelma.deaguiarpertinhez@unipr.it)

^2^ Department of Translational Medicine, University of Ferrara, Ferrara, Italy; AT: [anna.terrazan@unife.it](mailto:anna.terrazan@unife.it); PA: [pietro.ancona@unife.it](mailto:pietro.ancona@unife.it); NB: [nicoletta.bianchi@unife.it](mailto:nicoletta.bianchi@unife.it); FB: [federica.brugnoli@unife.it](mailto:federica.brugnoli@unife.it); VB: [valeria.bertagnolo@unife.it](mailto:valeria.bertagnolo@unife.it); SV: [s.volinia@unife.it](mailto:s.volinia@unife.it)

^3^ Department of Neuroscience and Rehabilitation, University of Ferrara, Ferrara, Italy; CMB: [bgc@unife.it](mailto:bgc@unife.it)

**Table S1.** Enriched metabolites derived from the heatmap analysis (mean scores >1).

| **Hbcx9** | 4-aminobutyrate  adenosine  betaine  glucose-6-phosphate  GTP  hypoxanthine  IMP  myo-inositol  N-acetyl-L-aspartate  NADP^+^  pantothenate |
| --- | --- |
| **Hbcx39** | AMP  betaine  glucose-6-phosphate  IMP  N-acetyl-L-aspartate  niacinamide  pantothenate  sarcosine  UMP  uridine |
| **MDA-MB-231** | fumarate  glutamic acid  guanosine  isocitrate  malate  nicotinate  O-phosphocholine  uracil  uridine |
| **MDA-MB-436** | 4-aminobutyrate  acetate  ADP  ATP  β-alanine  caprate  choline  formate  glycerol  methanol  NAD^+^  nicotinic acid adenine dinucleotide  pantothenate  proline  pyruvate  taurine  UDP-N-acetyl-D-glucosamine |
| **MCF-7** | 2-oxovalerate  alanine  aspartic acid  cystathionine  fumarate  glucose-1-phosphate  glutamate  histidine  isoleucine  leucine  lysine  malate  methionine  O-acetylcarnitine  phenylalanine  serine  threonine  tryptophan  tyrosine  UDP-glucose  UDP-N-acetylglucosamine  valine |
| **T-47D** | 2-oxobutyrate  AMP  arginine  asparagine  GSH  GTP  hypoxanthine  IMP  lactate  leucine  proline  serine  sn-glycero-3-phosphocholine  succinate |
| **SK-Br-3** | 2-oxoglutatate  2-oxovalerate  3-methyl-2-oxovalerate  alanine  arginine  asparagine  creatine  cytidine  glycine  nicotinurate  O-acetylcarnitine  O-phosphoethanolamine  pyroglutamate  serine  xanthine |


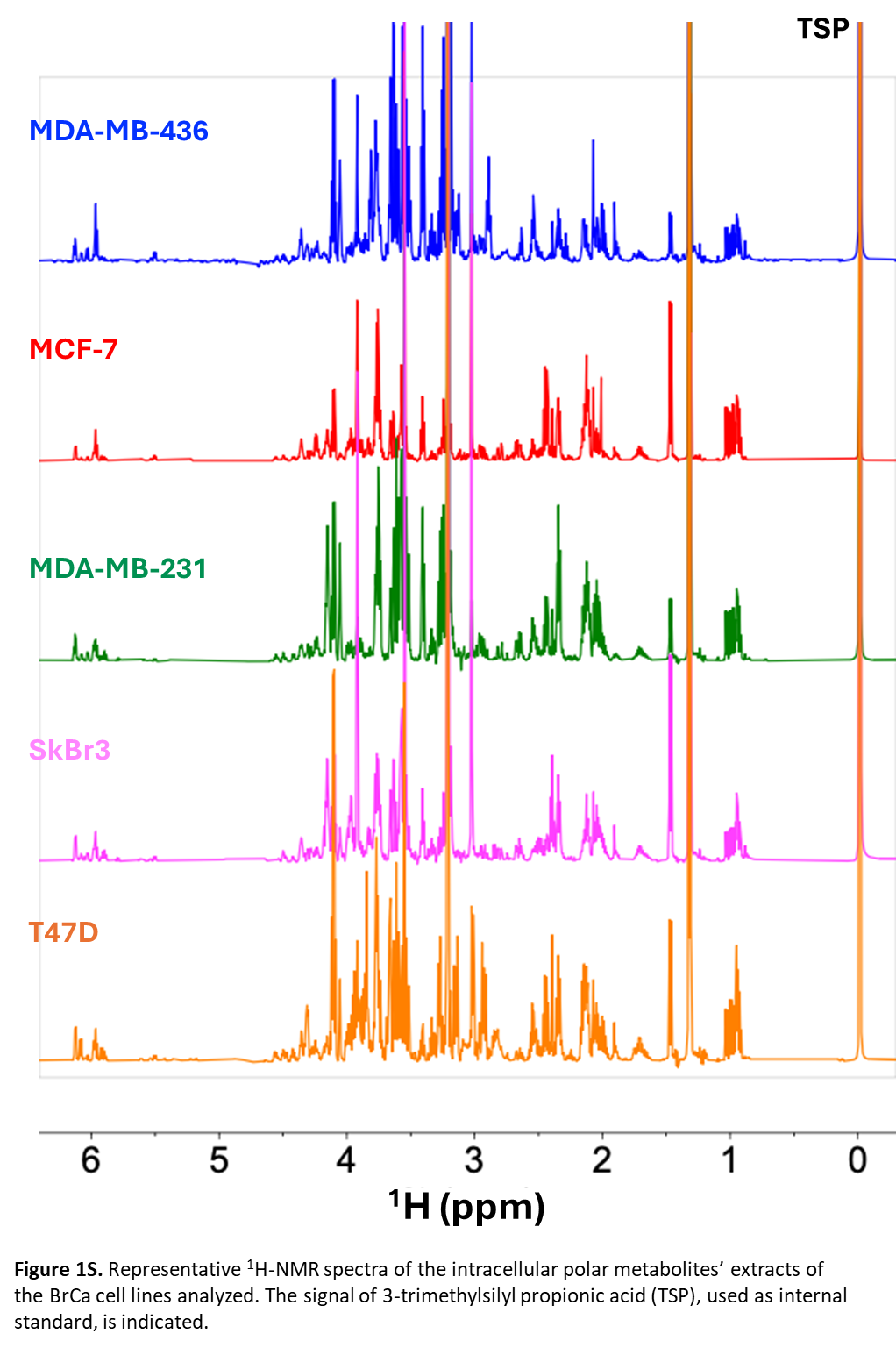


**Figure 1S.** Representative ^1^H-NMR spectra of the intracellular polar metabolites’ extracts of the BrCa cell lines analysed. The signal of 3-trimethylsilyl propionic acid (TSP), used as internal standard, is indicated.


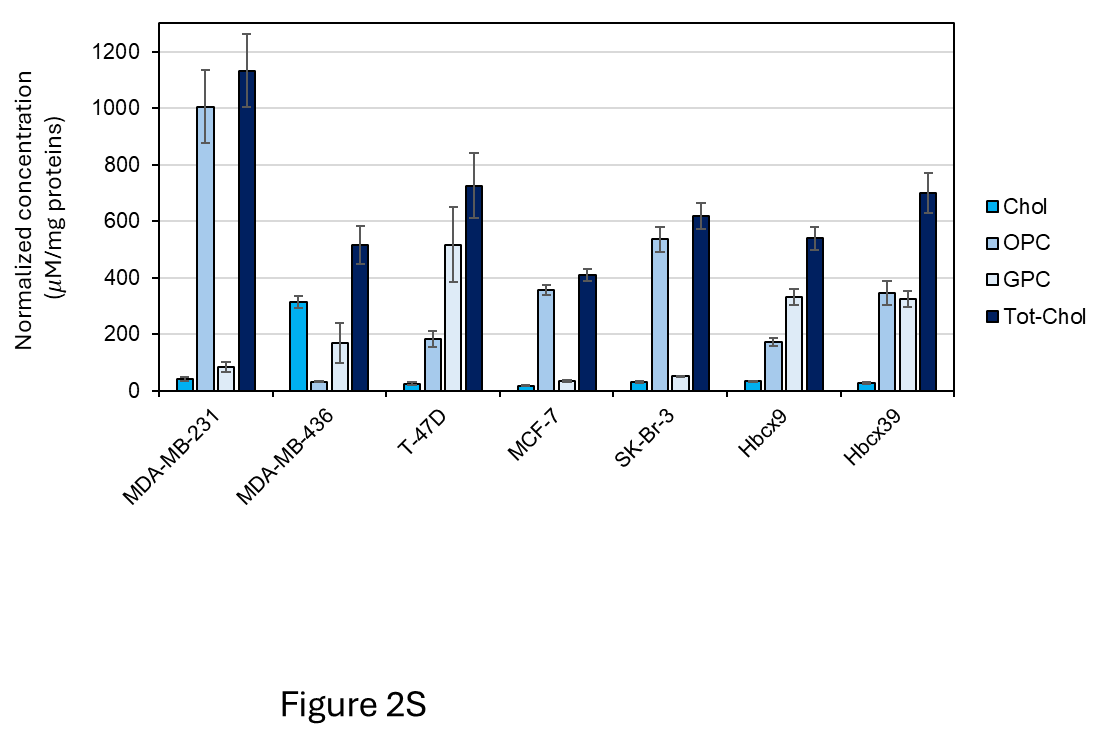


**Figure 2S.** The levels of choline compounds in the selected BrCa cell lines. Chol. - choline, OPC – O-phosphocholine, GPC – glycerophosphocholine, Tot-chol – total choline
